# Transactions of the Gynæcological Society of Chicago

**Published:** 1888-08

**Authors:** 


### SOCIETY REPORTS.

### TRANSACTIONS OF THE GYNÆCOLOGICAL SOCIETY OF CHICAGO.

REGULAR MEETING, MAY 25, 1888.

The President, Henry T. Byford, M. D., in the Chair.

Professor E. C. Dudley presented specimens of CARCINOMA UTERI AND A DERMOID CYST.

He said: I have two specimens that do not belong to the series of last year's operations, and consequently would like to present them before giving that series.

The first, a uterus removed for carcinoma; and the second, a dermoid cyst. When I first saw the woman from whom this uterus was taken, I found upon the posterior lip of the somewhat enlarged cervix a suspicious patch, so to speak, just posterior to the os externum, about the size of a five-cent piece, and being under the impression that it was probably a cancer, I excised a small portion of it for examination. Dr. Billings, after microscopic examination, pronounced it carcinoma. The uterus was freely movable, and the disease seemed to be confined entirely to this point. This uterus was removed through the vagina, at St. Luke's Hospital, about three weeks ago. No ligatures or sutures were employed in its removal, the hemostasis being effected by pressure forceps. Without hurrying or making an effort to hurry, I had no difficulty in getting the uterus out in about twelve minutes. Some additional time was required in securing the smaller vessels; it was an unusually hæmorrhagic case. When the operation was completed, the vagina was filled with forceps, about sixteen in all, of which the least important were removed in twenty-four hours, the more important in forty-eight, and the most important, on the broad ligaments, in seventy. The patient seems securely convalescent.

An interesting feature of this case is the early recognition of the disease; indeed, we rarely see carcinoma of the uterus in its incipiency, but, on the other hand, the disease has often advanced too far to give promise of permanent cure by hysterectomy. A case more favorable than this for cure could scarcely be imagined.

The next specimen is from a nullipara, twenty-two years of age, married two years ago. She was recently sent to me by Dr. Charles Gilman Smith. Five days ago I removed this tumor, that, as you see, is a dermoid cyst. There is a piece of bone, an abundance of hair, and a very great quantity of fat. The other ovary, which I have here, is cystic, considerably enlarged, and shows the corpus luteum of menstruation.

A peculiar feature of this case was the immense size, length, and diameter of the two Fallopian tubes; they were very œdematous, showing an obstruction in the circulation somewhere. The fluid of this tumor was very thick, ran through the trocar very slowly, and consequently some of it found its way into the abdominal cavity, and therefore, although there were no adhesions, I introduced a drainage-tube that was removed this morning, having discharged quite a large quantity of bloody serum during the three or four days after the operation. The patient gives every evidence of making an uncomplicated recovery.

This patient recovered.

The President showed the following specimens:

MULTIPLE SUBSEROUS FIBRO-MYOMATA OF THE UTERUS.

This specimen is a typical example of multiple subserous fibro-myomata of the uterus, ranging from the size of a small pea to that of a cocoanut. The enlarged ovaries, with the normal tubes, are hanging high up on either side. Here is a large mass, nearly the size of the fist, that projected down to the pelvic floor, and that had been felt per vaginam as a tumor beside the cervix. It was undergoing calcification. The patient, Mary N., was a negro virgin, thirty-two years of age, who had not menstruated until her twentieth year. Five years ago, a physician diagnosed a tumor of the intestines by external manipulation. The indications for removal were the failure of other treatment, uninterrupted growth, severe and ever increasing attacks of pain, and a location which would have rendered its further development dangerous and its future removal one of extreme difficulty. The menses, although more abundant than formerly, had been coming less frequently of late. This would seem to signify a falling off in activity of uterine and ovarian function, and a final cessation of growth—a result I have often seen in these cases of multiple subserous growths. Had the location and relations of this tumor been different, its removal would never have become a necessity. The broad ligaments and bladder were carried up so high, and were so closely adherent to the mass, that the prevention of excessive hæmorrhage during the formation of the pedicle was difficult, and made the operation long and laborious. After partial separation of the bladder and broad ligaments, a temporary elastic ligature placed around the middle of the tumor, and above the part where I had to work, was of great value in diminishing its vascularity and checking the profuse venous oozing. The figure drawn by Dr. Munde, on page 303 in the March number of the *American Journal of Obstetrics* of this year, represents almost exactly the relations of this tumor, except that the bladder is not shown. After separating the bladder sufficiently, I made an incision over the posterior surface of the pelvic growth, enucleated it rapidly and easily, and threw another elastic ligature around the uterus, at about the level of the internal os. The stump was fixed to the abdominal walls according to Hegar's method, except that I drained the lower end of the wound and surface of the bladder by means of a strip of iodoform gauze, left for three days; and also the bed of the enucleated tumor by a small glass tube above the stump, left for five days. I introduced the pins about half an inch above the elastic ligature, in order to let the cervix down as low as possible. Plain absorbent cotton was used as a dressing to the stump, after Bantock's manner, causing it to become dry and horny, and thus entirely harmless. She recovered without an unfavorable symptom, and took no medicine except a laxative.

UTERUS REMOVED FOR CARCINOMA OF THE CERVIX.

I have here a uterus, with a broad rim of vagina attached, that was removed for cervical carcinoma; also the left ovary, which was four times its natural size, and imbedded in lymph, but not carcinomatous.

The patient, Mrs. O. B., is 25 years old, and has two children, the younger one but two years old. As the posterior wall was infiltrated up to the internal os, and the uterus more rigid than usual in its attachments, I started out to make an exploratory incision in the cul-de-sac of Douglas, and expected, if I found evidences of extension of the disease beyond the uterus, to close the incision and wait two or three weeks before attempting a palliative operation. I dissected and tore my way up between the uterus and rectum, but could not get into the peritoneal cavity. A fibrous band, extending from the posterior cervical wall backward, was in the way. Feeling confident that the uterus, and perhaps all of the disease, could be safely taken out, I carried the vaginal incision completely around the cervix, dissected off the bladder, and pulled the fundus forward into the vagina. The fibrous band that connected the uterus with a knuckle of intestine was dried and cut and the cul-de-sac opened from above, the broad ligaments clamped with hemostatic forceps, and the uterus cut out between them. The ovary was then peeled out of its bed of lymph in the left lateral sacral peritoneal pouch, ligated and cut off. The ligatures, which were left long enough to protrude at the vulva, came away in about ten days. The peritoneal wound was left open, and the vagina plugged with iodoform gauze. The forceps were removed in twenty-eight hours, and the tampons in four days. The recovery has so far been uneventful and uninterrupted, and without any medication except a single dose of morphine and the usual laxatives.

Martin, Fritsch, Leopold, and others, have adopted set methods of procedure for vaginal hysterectomy, from which they seldom vary, except in unimportant particulars. Their skill may enable them to do so. My experience with six cases has, however, led me to believe that the safest way for the patient is to vary the operation to suit the case. For instance, in this case I could not have ligated the broad ligaments before anteverting the uterus without working in the dark and greatly increasing the danger. In my third case the uterus was so broad that I could not isolate the broad ligaments until I had retroverted the uterus. In my second case the vulva and vagina were so small, and the tissues so rigid, that I could not readily antevert or retrovert, nor could I draw the organ down so as safely to apply ligatures, hence I applied three forceps on each ligament, cutting the tissues clamped by one pair before applying the next one above. In the first, fourth, and fifth operations I applied ligatures from below upward, scarcely touching or seeing the peritoneum except on the stumps and on the uterus.

The cases all recovered, and it seems to me of much less importance whether or no sutures or forceps are used, or whether the uterus is anteverted, retroverted, or merely drawn down, or whether half an hour or two hours is consumed, than that the operation be properly, carefully, and completely done. To choose the most direct method for the particular case, to handle the tissues carefully, to make sure against hæmorrhage, and to provide an exit for the discharges, makes the best operation and will give the best results.

### THE UTERINE APPENDAGES REMOVED FROM TWO CASES BY VAGINAL SECTION.

I wish also to present specimens from two cases of vaginal section, made four weeks ago for the removal of the uterine appendages. You will notice that the organs are not mutilated, and present the same appearance as if removed by abdominal section. In one case the tube was dilated with bloody serum, and resembled an intestine so closely that I made a thorough digital exploration before I ventured to ligate it. The ovary is about the shape of a slice of bread, and fully four times its natural size. The ovary and tube were adherent to each other and to the posterior surface of the broad ligament. Both patients are making a good recovery.

Professor C. T. Parkes: I expected to have two specimens as the result of vaginal hysterectomy with me to-night, but they have not come, and I will refer to them only so far as Dr. Dudley's specimen is concerned. In both cases the manifestation of the disease was not great. In one, at least, the diseased point did not cover a space any larger than in the case shown by Dr. Dudley, and in both of them sections were made and submitted to microscopic examination, and they were shown to be carcinomas. The uterus was removed by vaginal section. I was interested in what Dr. Dudley said about the time at which the cancer was removed. He said that in all the cases of complete removal of the disease, those cases that were removed as early as the one shown would be the most promising cases. I remember very distinctly, in both of these operations, on the side in which the disease showed itself the section of the broad ligament with the scissors was not as easily made as on the other side, and when removed the density of the tissue was greater on that side than on the other, creating in my mind the suspicion that some absorption had taken place, although it was not positively detectable; so these cases I shall watch with interest to see if there is any return of the manifestations.

I was somewhat surprised to hear Dr. Dudley say that in this operation, which was done so readily and rapidly, he was compelled to use sixteen forceps. I have done this operation half a dozen times, and in none have I been required to apply more than three forceps, although the bleeding was in each controlled by hemostatic forceps. In these last two cases, the specimens of which I will show at some future time, but two forceps were used; they were long forceps, and embraced the whole length of the broad ligament fairly and squarely, and there was no hæmorrhage of any consequence. Both patients have passed beyond the necessity for further attention, and their recovery is established so far as the operation is concerned.

One point with reference to the removal of the fibroid uterus and the use of the elastic ligature. I remember in one of my cases of removal of the fibroid uterus, of using the elastic ligature as a temporary application to control hæmorrhage. In one case where I applied the ligature—and I speak of it as a warning as to the care required in its use—the section was made low down, and there was very little stump to take hold of after the uterus was removed; I congratulated myself upon the ease with which it was done, and, in the midst of my congratulations, the ligature slipped over the end of the stump, and the stump was at the bottom of the pelvis, and the woman lost a good deal of blood before it could be secured.

I had the honor of reporting a case of successful removal of a good-sized tumor about two years ago. Two or three days ago a lady came into my office, perfectly well so far as her general health is concerned, but with an open sinus in the abdominal wall and through the vagina, with the history that some months ago an abscess formed in the line of the incision, and from the opening she got a ligature. You will remember that in reporting that case I said I had had five cases of removal of fibroid tumors, and the first three died. These three were done by the extra-peritoneal method, and I concluded that was not my forte, and the next case, which was this one, I treated by the intra-peritoneal method, and the woman got well promptly, and until a few months ago was entirely well, until these ligatures came away of their own accord. The other case was also done intra-peritoneally, and she is perfectly well and doing her ordinary work now.

Dr. John Bartlett: Dr. Parkes refers to the difficulty he got into on account of the slipping of the rubber ligature. He should have indicated the way in which he helped himself out of it. For a moment he vainly sought for the lost pedicle lying deep in the pelvis in a pool of blood; he then thrust the handle of an instrument into the vagina and thus lifted the stump of the cervix out of the pelvis within easy reach.

Dr. E. C. Dudley: It is possible that, as far as hemostasis is concerned, the forceps may be safely removed after twenty-four hours, but there are two reasons for leaving them longer. First, the wound secretions, always abundant during the first two or three days, find their way by continuity of surface along the track of the forceps out through the vagina; the forceps, therefore, very effectively facilitate drainage. Second, the tissues grasped in these forceps are certain to slough, and this being the case the sooner the sloughing tissue comes away the better. Now, the longer the forceps are left the longer the tissues will be compressed and the more quickly will they slough, and if left two or three days, the tissues in their grasp will come off almost as soon as the forceps are removed, or the forceps may even drop off without being removed at all.

It is better ordinarily not to antevert or retrovert the uterus, because if a carcinomatous cervix is thereby forced into the peritoneal cavity, carcinomatous or septic infection may result. Moreover, when the uterus is anteverted or retroverted, the cervix being in the peritoneal cavity and the corpus being in the vagina, you have the larger portion of the uterus in the vagina, where it is an impediment to the operator.

As soon as the opening is made it is well to force a sponge through it into the pelvic cavity, then another and another. They prevent the formation of blood clots, which it might be difficult to remove. When the operation is done withdraw these sponges, and the toilet of the peritoneum is made. A string attached to each sponge keeps it from working its way beyond the reach of the finger.

I shall never again use the vaginal tampon of iodoform gauze as a last step in the operation. The vagina is an excellent drainage tube if left open, but the tampon impairs its efficacy as such. An antiseptic dressing may be placed over the vulva around the handles of the forceps; if any tampon at all is to be used in the vagina it should be placed between the anterior wall of the vagina and the forceps, not between them and the posterior wall where it would be doubly certain to obstruct the outflow of the wound secretions.

Professor E. C. Dudley read the following paper, entitled A Year's Work in Abdominal Surgery.

The list of seventeen cases which I now report includes all of my work in abdominal surgery in 1887. Eight operations were for the removal of the uterine appendages, seven for the removal of ovarian and parovarian cysts, one for the removal of the uterus through the vagina, and one for the incision and drainage of a pelvic monocyst.

The eight patients from whom the uterine appendages were removed had, in every instance, suffered from recurring pelvic inflammations, which had rendered their lives miserable, for which other means of relief had apparently been exhausted, and for which this operation was a final resort. The results of these operations cannot be as satisfactorily reported now as they might be at a later date.

Cases 2, 8, 14, and 17 have been apparently cured.

Cases 3 and 12 were of nervous, neuralgic patients who had suffered for many years from disorders of nutrition, dyspepsia, dysmenorrhœa, pelvic pains referable to the region of the ovaries, particularly the left ovaries, which were prolapsed, and from various other disturbances which go to make up the symptom group of hysteria. These have been improved, but the improvement has been chiefly confined to the relief from pelvic pain and dysmenorrhœa. It is too early to predict results relative to the nervous aspects of these two cases.

Case 4 was not materially relieved until after the shortening of the round ligaments for a retroversion, which persisted after the removal of the appendages. I am informed that she is now very materially improved, if not cured. Case 5 has passed from my observation, and results cannot therefore be given.

In Case 8, each tube contained not less than four ounces of pus. The ovaries were cystic and enlarged; no adhesions. One tube was brought up into the wound, and its contents drawn off by means of a small trocar, before the ligature was applied. The other was ligatured and removed intact. The tubes were enormously distended, and I think would have burst before long had they not been removed. The peritoneum in the region of the appendages was studded all over with small pearly

| Number. | Residence. | Medical Attendant. | Age. | Married or Single. | Disease. | Operation. | Drainage. | Date. | Recovered. | Died. |
|---|---|---|---|---|---|---|---|---|---|---|
| 1 | Danville, Ill....... | Dr. R. W. Gillette. | .. | M. | Ovarian tumor.................. | Removal of left ovary......... | No. | February | R. | .... |
| 2 | Chicago, Ill....... | Dr. Charles Gilman Smith. | 33 | M. | Recurring pelvic inflammation ; prolapsed left ovary ; extreme retroflexion and hypertrophy. | Removal of uterine appendages | No. | May 7 | R. | .... |
| 3 | Grand Rapids, Mich. | Dr. E. C. Dudley.. | 27 | S. | Recurring pelvic inflammation ; prolapsed ovary ; salpingitis. | Removal of uterine appendages | No. | June 1 | R. | .... |
| 4 | Chicago, Ill.. ..... | Dr. John W. Koehn. | 29 | S. | Recurring pelvic inflammation ; prolapsed ovary. | Removal of uterine appendages | No. | July 2 | R. | .... |
| 5 | Assumption, Ill.... | Dr. Rivard........ | 34 | M. | Inflam. of uterine appendages ; recurring peritonitis; retroflexion. | Removal of uterine appendages | No. | July 2 | R. | .... |
| 6 | Rock, Iowa... .... | Dr. Charleton..... | 18 | S | Ovarian cyst ; exten. adhesions.... | Removal of left ovary......... | Yes. | July 5 | R. | .... |
| 7 | Sycamore, Ill...... | Dr. W. W. Bryant. | .. | S. | Parovarian cyst.................. | Removal of left ovary......... | No. | July 20 | R. | . |
| 8 | Thorndyke, Mass.. | Dr. G. H. Felton.. | 32 | M. | Double pyo-salpinx ; cystic ovaries. | Removal of uterine appendages | No. | Sept. 6 | R. | .... |
| 9 | Princeton, Ill...... | Dr. A. H. Thompson. | 30 | S. | Pelvic monocyst.................. | Abdominal section ; drainage.. | Yes. | Sept. 26 | R. | .... |
| 10 | Burlington, Vt..... | Dr. Frank Billings. | 38 | M. | Right parovarian cyst; cystic ovaries; salpingitis. | Removal of both tubes and ovaries. | No. | Oct. 13 | R. | .... |
| 11 | Chicago, Ill....... | Dr. G. W. Webster | 38 | M. | Ovarian cystomata ; universal adhesions. | Removal of both ovaries...... | Yes. | Oct. 17 | R. | .... |
| 12 | Grand Rapids, Mich | Dr. E. C. Dudley.. | 28 | S. | Recurring pelvic inflammation; prolapsed ovary. | Removal of uterine appendages | No. | Oct. 28 | R. | .... |
| 13 | Sandwich, Ill...... | Dr. V. V. Vermilye | 23 | M. | Parovarian cyst.................. | Removal of left ovary......... | No. | Nov. 1 | R. | .... |
| 14 | East Saginaw, Mich | Dr. Thrall........ | 37 | M. | Inflammation of uterine appendages ; universal adhesions. | Removal of uterine appendages | Yes. | Nov. 4 | R. | .... |
| 15 | Chicago, Ill....... | Dr. M. L. Scudder. | 36 | M. | Ovarian cyst ; exten. adhesions. | Removal of left ovary......... | Yes. | Nov. 16 | R. | ... |
| 16 | Sturgeon Bay, Wis. | Dr. Sibree......... | 51 | M. | Sarcoma of uterus; rectal adhesions. | Vaginal hysterectomy..... ... | No. | Nov. 19 | R. | .... |
| 17 | Sturgeon Bay, Wis. | Dr. Sibree. ....... | .. | M. | Inflammation of uterine appendages | Removal of right ovary and tube | Yes. | Dec. 24 | R. | .... |

NOTE.—One of the above operations was performed at a private residence; fifteen at St. Luke's Hospital; and one at the Presbyterian Hospital. Fourteen, though in hospital rooms, were private patients.

NOTE.—This list of seventeen consecutive recoveries might be increased to twenty-one by going outside the year 1887.

points, giving evidence of miliary tuberculosis; no ascites. Dr. Frank Billings, upon microscopic examination of the contents of the tubes, found the bacillus tuberculosis. This is contrary to the statement of an English ovariotomist, who declares that tuberculosis does not exist in the tubes. No drainage was used; perhaps drainage might have been desirable on account of the tubercular disease in the peritoneum.

In Case 17, only the right ovary and tube were removed, the other being entirely absent—a condition which has been observed in other cases. This woman, however, had borne children, and contrary to rule in such cases, the uterus was entirely symmetrical, the left side having been as perfectly developed as the right. At the left horn of the uterus, where the tube should have joined it, there was a slight protuberance, indicating a very rudimentary tube, and at the point of this protuberance, a little depression could be seen, but whether there was a connection between this depression and the interior of the uterus I did not determine. The ovary and tube which were removed were extremely adherent; the ovary was cirrhotic and had been the seat of pain for years.

Of the seven ovariotomies, four were for ovarian and three for parovarian cysts. They illustrate both the gravity and the simplicity of these operations. The parovarian cysts were easy of removal; the others were adherent, and two of them presented difficulties which rendered their removal almost impracticable.

In Case 11, the tumor was so intimately adherent throughout its entire surface that I was unable to break up the adhesions in the usual way, but was obliged to split the cyst-wall, leaving what might be called the capsule of the cyst in the abdomen, stitching it to the abdominal wound. The layers of the cyst-wall were so intimately connected that the greatest difficulty was experienced in separating them. The torn surfaces bled so profusely that I used Mikulicz's drainage, packing the cavity with iodoform gauze, leaving it in for twenty-four hours, and then substituting the drainage-tube. After a long, tedious convalescence, the patient was discharged, having a fæcal fistula at the lower extremity of the wound. Whether this fistula resulted from some damage done to the intestine in the operation, or from the pressure of the glass drainage-tube, or from an ulcerated condition of the lower bowel, which had been recognized previous to the operation, I do not know. She had been a victim of epilepsy, which for a number of months after the operation was in abeyance, but which has now reappeared and of which she will probably die.

In Case 6, the adhesions were also very extensive; not less than a square foot of surface was exposed in separating them. After breaking up some very extensive parietal adhesions on the right side, the hæmorrhage was quite profuse and from a hundred points, and not controlled by the ordinary isolated ligatures. Hemostasis was finally secured by passing a number of silk sutures, half an inch apart and parallel to one another, deep down beneath the bleeding surfaces, and tying them tightly. This method seems preferable to the actual cautery. It is rapid and effective. The patient did well for three weeks and seemed to be surely convalescent, when she came near dying of septicemia consequent upon several hypodermic sloughs, hypodermics having been given at the time of the operation for an alarming heart failure.

In Case 1, a croupous pneumonia developed immediately after the operation, from which the patient narrowly escaped a fatal result.

The vaginal hysterectomy was for sarcoma uteri, and has previously been reported to this society. The patient, I understand, continues in good health.

The case of incision with drainage was unlike anything I had ever seen. The cyst-wall was very thin, was opened directly without invading the abdominal cavity, and so far as I was able to determine, was intimately adherent all around, except perhaps deep down in the pelvis and on its posterior surface. I could feel the ovaries and uterus through the cyst-wall. Lawson Tait describes a variety of abdominal cyst in many respects similar to this.* He reports six cases, all occurring in young women between the ages of sixteen and twenty-six. Before operation they appeared to be parovarian cysts. Upon opening the abdomen, were found intimate adhesions between the cyst and peritoneum, limpid fluid, cysts lined with epithelium, smooth glistening surface; the uterus and ovaries could be felt through the cyst-wall, were apparently healthy and independent of the cyst. The tumors were therefore neither ovarian nor parovarian. Tait is disposed to refer them to a distinct class of pathological cysts. His impression is that they are formed by dropsical distention of an ovule which had not become impregnated, but which, having dropped into the peritoneal cavity, had there become attached and developed.

An examination of the contents of this cyst gave the following results:

*Report of Analysis* of fluid from abdominal cyst opened by Dr. Dudley at St. Luke's Hospital, September 26, 1887. A sample of the fluid containing about three fluid ounces was taken.

Upon inspection the fluid appears clear, translucent, and contained no (macroscopic) sediment. Color, amber; odor, none; reaction, neutral; specific gravity, 1.008.

Chemical examination showed albumen present in large quantity (nitric acid, dilute, and heat test), so that the mixture coagulated into a semi-solid mass.

Another portion diluted four times by distilled water showed, after applying the acid and heat, shaking to break up the coagulum, and allowing to settle for twenty-four hours, a precipitate filling one-third the bulk of the mixture.

Applying Francklyn's method of reduction (*Journal American Medical Assoc.*, April 4, 1885), it is found that the original fluid contains about .026 by weight of albumen.

Tests for urea, uric acid, phosphates, and peptones were applied with negative results in each instance.

Microscopical examination of twelve slides revealed no sediment except a few particles of amorphous material (probably extraneous). H. H. FROTHINGHAM.

43D STREET AND LAKE AVE., OCTOBER 3, 1887.

*Preparatory Treatment.*—Unless there was some special indication to the contrary, the preparatory treatment was short and simple, occupying not more than two or three days, as follows: A cathartic about forty-eight hours before the operation; repeated vaginal douches of hot castile soap suds, with thorough cleansing of the external genitalia and of the entire abdominal wall, especially of the umbilicus. One or two general shampoo baths or if practicable a Turkish bath with a lather shampoo of the hair. The hour for operating has been nine o'clock in the morning, a cup of beef-tea having been given two or three hours before. Previous to the day of the operation diet is not restricted or modified.

*Antisepsis.*—Antiseptic drugs as a rule were not used in direct connection with the operation. They were employed for the purpose of rendering hands, instruments, and patient surgically clean, and then thoroughly washed off with water which had been sterilized by filtering and thrice boiling; that is, antiseptic drugs were not brought in direct contact with the wound. Sponges which had been kept in weak solutions of sulphurous acid, carbolic acid, or corrosive sublimate were never used until these drugs had been thoroughly washed out with sterilized water. Indeed, everything that was to be in direct connection with the operation was treated in this manner. Fumigation of the patient's room has only been done when it had previously been occupied by several cases or by a suspicious case. Sometimes,

* "The Pathology and Treatment of Diseases of the Ovaries." Fourth edition, page 184. William Wood & Co.

as a matter of ceremony, a little iodoform was sprinkled over the wound before the dressings were applied, but it smells bad and may do harm by exciting or keeping up nausea.

If, in the toilet of the peritoneum, there be blood, oozing points, or pus, or if these be even suspected, I wash out the abdomen freely, putting in quarts or gallons of water, and when in doubt whether this should be done, I remove all doubt by doing it.

The same rule applies to drainage. If in doubt, always drain. I have recently lost a patient whom drainage might have saved. The adhesions were extensive, but the abdomen being perfectly dry, I closed without drainage. She did badly for the first thirty-six hours. I reopened; there had been no hæmorrhage, but the abdomen contained an abundance of bright red serum. If this had not been allowed to accumulate at all, the result might have been different.

The glass drainage-tube is always preferred. The tubes kept in the shops are too large. I have had some made, of the diameter of lead pencils, having the shape of test tubes, with many small perforations the size of a pin head at the closed end. Two or three of these tubes may be introduced if desired. They do no harm; they can be removed if nothing comes through, and the openings immediately close, and they carry off the bloody serum or other fluids as efficiently as tubes of large size. Drainage not only prevents septic infection, but by keeping the abdomen dry serves as a hemostatic, as moisture favors hæmorrhage. It is important that the perforations at the end of the drainage-tube be quite small, otherwise portions of omentum are apt to work themselves through and make trouble in the removal of the tube. I have recently had two such cases; in one the tube was nearly half full of omentum which had worked its way through an opening only one-tenth of an inch in diameter. This annoyance may be in a measure prevented by giving the tube a turn or two whenever the dressings are opened.

*Medication and Diet.*—The cases included in this report have recovered with very little medicine, some without any at all. Opium has been used very exceptionally. A patient who begins to take opium for pain after abdominal section ordinarily continues to have the pain and to require the opium; but if the drug be withheld, the pain generally subsides.

Case 11 strikingly illustrates the advantage of a non-opium treatment. Abdominal tenderness and distention, and other signs of peritonitis appeared soon after the operation; it became essential to relieve the distention by evacuation of the bowels; soap and turpentine enemata were inadequate, and the movement was not without difficulty secured by means of calomel and soda, whereupon the peritonitis subsided. Had the secretion been locked up under the influence of opium, the peritonitis would have probably extended and I fear with fatal result.

It is, perhaps, not too much to say that the modern treatment of peritonitis by catharsis, judiciously employed, is sound. Not less than half of my patients after abdominal section have a cathartic before the end of the third day; the others are usually treated with copious enemata of stiff soap suds in which a teaspoonful of turpentine to the quart has been thoroughly mixed. Upon the least suspicion of distention an action of the bowels should be secured.

During the first twenty-four hours no food whatever is given; only a little hot water, or ginger ale, or possibly champagne. On the second day a little barley water is cautiously given, soon to be followed, if there is no disturbance or nausea, with half-teaspoonful or teaspoonful doses of milk, repeated occasionally and increasing in quantity, as the patient gives evidence of being able to bear it.

*The Staffordshire Knot.*—Three years ago I saw Mr. Tait apply the Staffordshire knot. In the first case, after my return, I attempted to apply it and the patient died of hæmorrhage. The next year I saw Mr. Tait operate fifteen or twenty times, and particularly observed his method of applying this knot, and since then have used it invariably, and consider it, generally speaking, the best ligature. A distinguished surgeon in New York has lost a number of patients from hæmorrhage with the Staffordshire knot, and has discarded it as dangerous. Indeed, a number of operators have had most unpleasant experiences in its use.

The secret of Mr. Tait's success lies in a single maneuver. After the pedicle has been transfixed, the loop drawn through and brought over to the point of transfixion and placed between the two free ends of the ligature, these latter are held firmly between the thumb and finger of the left hand close to the point of transfixion. Then with the right hand he catches each free end separately, and draws the ligature perfectly tight, and while the thumb and finger of the left hand still hold the thread at the point of transfixion to prevent the ligature from slacking again, the operator, with his right hand, aided by the assistant, makes a hard knot.

An additional precaution to prevent the ligature from slipping may be wisely observed by transfixing at two points, first forcing the loop through at the juncture of the Fallopian tube and uterus in a direction from the operator, then carrying it along on the further side of the broad ligament and drawing it through again in the direction of the operator, transfixing at the hilum of the ovary. The loop may then be drawn over the tube and ovary and that portion of the broad ligament which it includes and tied as already described. This modification of the Staffordshire knot, which, I am informed, Mr. Tait also occasionally employs, makes hemostasis doubly certain, and is to be preferred on this account.

A word about the silk. The great annoyance which every operator has experienced in breaking a thread at a critical moment, while attempting to apply a firm ligature, is sufficient proof that the silk ordinarily sold by instrument makers is generally inferior and often worthless. A variety of twisted silk, known as "Chinese Grass," may be found at the fishing-tackle shops. For surgical purposes it is unexceptionable, inasmuch as it has the qualities of absolute purity and great strength.

*The Arrest of Menstruation.*—One of the chief objects in the removal of the uterine appendages, in a great majority of cases, is to arrest menstruation; in other words, if menstruation be not arrested, the operation in very many cases fails. In the early history of the operation the ovaries alone were removed, or the ovaries and a part of the tubes. It was found in some cases that menstruation continued as before or increased. Then the tubes began to be removed also, and the complete arrest of menstruation was more frequent. It was further found that if the tubes were removed entire, close to the uterus, menstruation was almost always arrested, and that in many cases which were thought to be exceptions, the tubes in reality had not been entirely removed. Oftentimes a small knuckle of tube was discovered to have been left, and to be so closely adherent to the uterus that it escaped notice. The removal of this knuckle has been known to arrest menstruation. Reasoning from these facts, it was concluded that the tubes really have more to do with menstruation than the ovaries.

Contrary to this idea, Dr. Arthur Johnston, of Danville, Ky., in a conversation with me several months ago, said that the true explanatlon of these facts might involve an entirely different conclusion. There is a little plexus of nerves in the broad ligament, in the angle formed by the uterus and Fallopian tube. When the tube is entirely removed, this plexus of nerves is entirely removed also, and on this account it may be that menstruation ceases, rather than on account of the removal of the tubes.

If this be true, it is a fact of immense value. Possibly a ganglion may be found in this region, and it may follow that the removal of this plexus alone, without reference to the ovaries and tubes, may arrest menstruation. This specimen from Case 2 illustrates the plexus of nerves, which is easily recognized by the naked eye.

*The Incision.*—The opening into the abdomen has, in most instances, been short. Surprising as it may seem, it is sometimes easier to perform difficult manipulations in the abdomen through a small opening than through a large one. The large opening permits the intestines and omentum to rise up in the way of the operator, and to render inaccessible the field of operation. This is even true of large ovarian cysts with extensive adhesions. After the removal of the fluid, the lax abdominal wall permits the opening to be moved about to almost any part of the cavity. In many instances the short incision enables the operator to do his work with the minimum amount of operating, and for obvious reasons, therefore, with the minimum risk.

It is well, in closing the abominal wound, to tie the sutures with bow knots, leaving the ends long, in order to obviate the necessity of introducing new sutures, in case it becomes desirable, at any time, to re-open the wound.

*General Remarks.*—It has so happened that in almost all of these cases there has been a steam radiator under the window before which the operations were done, the patient's feet being toward the window. This insured a constant warmth of the feet during the operation, and perhaps has in some degree contributed to the freedom from shock.

In removing the appendages, the toilet of the peritoneum may be much facilitated by forcing a soft sponge down into the cul-de-sac of Douglas as soon as a tube and ovary is drawn up into the wound to be ligatured; two or three sponges may be required. If there is much oozing, they may be frequently changed. By this means the blood is immediately taken up by the sponges, and when these are removed the peritoneum is dry. Otherwise blood would find its way into the cul-de-sac and form a clot which might escape notice.

I have not brought the specimens, with the single exception of Case 2, because there is not very much of interest in the ordinary specimen. Every one presents specimens in abdominal surgery, and it has, therefore, ceased to be a luxury to look at them unless they are very remarkable.

Professor C. T. PARKES: I think Dr. Dudley is to be congratulated upon these interesting and successful cases, but I think the doctor will not have done all his duty until he has given us some of the snags he has met with in the shape of deaths. These cases are full of interest, but I have always found the cases that have died have been the ones from which I have learned the most. I have no doubt that will come in due time.

So far as my experience goes in the removal of the uterine appendages, in every case there has been found disease of the appendages or ovaries, there was either closure of the internal or external opening of the tube, some enlargement, or some disease of the ovaries themselves, which really pointed to the condition of the appendages as the cause of the trouble.

In the case Dr. Dudley reports of cyst with drainage, that had no connection with the uterus or ovaries, which, after an opening was made into the abdominal cavity and the finger introduced, the ovaries and uterus were felt perfectly normal, gave rise, in my mind, to the suspicion that, instead of being a cyst with a distinct and separate wall that could not be recognized or differentiated from the peritoneum, it was a case similar to one I have seen, which would come under the appellation of an encysted dropsy, where the inflammation of the peritoneal cavity had been of such a nature as to agglutinate the intestinal folds together, and formed a perfect roof to the cavity, and the fluid had gone on accumulating until the quantity of fluid had shown the external manifestations of a cyst; when cut into, the cavity was found to have no connection with the uterus. It is what Spencer Wells calls an encysted dropsy. I think it would be difficult to say that there was a true cyst-wall in a case of that kind, if no separation whatever could be found. The character of the fluid he mentions rather points to that condition.

I was exceedingly well pleased to hear the doctor speak of his experience with reference to antiseptic precautions; it agrees with my experience so far as abdominal work is concerned. In a case I reported a few years ago, the only case in which I had had much trouble in that series, and in which I carried out Lister's instructions, it gave me more trouble than all the rest, and I think it was from using too strong antiseptic applications, so I resumed the same course the doctor has indicated with regard to antiseptic precautions in abdominal cases. Several years ago, in the Chicago Medical Society, I took occasion to refer to the fact that I had tried the Staffordshire knot and it failed. I did an operation for the removal of the uterine appendages before a class at college, and desired to illustrate the Staffordshire knot. I thought I understood all about it, and when I had it applied, cut off the appendages and returned the stump to the abdomen, but to my astonishment the wound filled with blood, and I had quite a time to fish it up from the cavity and stop the bleeding which was going on freely. I recognized then the very fault the doctor has illustrated; the trouble was that I did not secure the pedicle at all in tying the knot. Recently, on a visit to New York, I saw Professor Pope do two laparotomies for uterine disease. In one of them I asked him to illustrate to me the application of the Staffordshire knot, and he did it in the manner which the doctor has illustrated, and showed me plainly where my fault had been in not pulling up the ligature on each side before the knot was tied.

So far as my experience goes, in many of these cases which require the removal of the appendages, there are no symptoms that can be fixed, so far as the physical examination is concerned, as coming from any disease of the uterus or appendages, but after the operation is done it often turns out that disease is found in the tubes.

There was one point I wished to speak of—the case of epilepsy relieved by an operation. I am associated with a gentleman who has had a great deal of experience in one of the insane asylums with epileptics, and he has found it to be a fact that no matter how small an operation may be done, and no matter where it is done, as a direct effect of that operation the patient is relieved of epileptic seizures for some time.

Dr. Henry T. Byford: I have had a great deal of experience with Tait's knot, but it has all been confined to one case. I pulled the threads tightly before tying, closed up an apparently dry peritoneal cavity, and lost my patient from hæmorrhage. The ligature had not held. As the method which I ordinarily employ has never failed me, I have not since felt inclined to try the more complicated varieties, which, although they may be as safe, are more difficult of application. I pass a double thread through the edge of the ovarian ligament and through the mesosalpinx near the Fallopian tube, thus tying up the ovarian ligament, Fallopian tube, and ovarian artery on one side, the rest of the broad ligament on the other, and then the whole stump *en masse* with the same threads.

With regard to after-treatment, I think that one of the most important recent advances has been the administration of saline laxatives in place of opiates; as soon as symptoms pointing to peritonitis, sepsis, or intestinal obstructions make their appearance, they must be given early.

Professor Knox: What is your object in getting antiseptics out of your ligatures and instruments?

Professor DUDLEY: Not having removed the cyst in Case 9, I am unable to prove that a cyst existed. I think Dr. Parkes would readily agree with me had he been present at the operation. Among those who saw the operation there was no difference of opinion.

Fumigation of an operating room is undoubtedly an element of safety, although it is practically impossible to sterilize the air of a room by this or by any other means, because, after fumigating, other air will come in. It is evident, however, that, under ordinary conditions, the danger of infection from the air is very small as compared with the danger from non-sterilized fingers, instruments, ligatures, sponges, etc. The object of washing instruments, sponges, and ligatures free of antiseptic drugs is to avoid the irritating influence which these drugs might exert if brought into direct relation with the operation.

The question has been raised relative to the removal of the appendages for the relief of dysmenorrhœa. In none of the cases reported was dysmenorrhœa the prime indication for the operation, although dysmenorrhœa was frequently one of a variety of morbid conditions, the pathological causes of which it was hoped the operation would remove. In Case 2 there had been dysmenorrhœa, but the operation was not done on that account, although I would not say that it never should be done in an extreme case, incurable by other means.

Professor JAGGARD: What was the indication for operation in this case of removal of the appendages? The specimens show no appearances of disease that I can make out.

Professor DUDLEY: This was a case of retroflexion, which I had failed for nearly two years to support by artificial means. The patient had suffered for several years from recurring attacks of pelvic peritonitis; the left ovary was prolapsed and was the seat of constant unremitting pain. Although urged to do this operation for a year, I had refused, and was only induced to undertake it after a long effort to give relief by other means. There was no element of hysteria in this case.

The patient had been a victim of more or less constant and acute pain; since the operation she has been free from pain. She had been obliged to spend a large part of each day in the reclining position; since the operation she requires the usual amount of rest at night, and no more. Her nutrition had suffered to an extreme degree from reflex gastric disturbances, and she was unable to take much food of any kind; soon after the operation digestion became normal, she could take all kinds of wholesome food, and the anæmia and emaciation promptly disappeared. She had been unable to walk or stand without great difficulty, or to endure the least fatigue; since the operation she has been able to swim, to walk, and to dance without limit. The patient has been a pitiable invalid; after the operation she became well.

In most of the other cases the appendages themselves showed more gross indications of disease, but in this case the removal of the appendages has given relief to a most distressing and most obstinate malady, which was just as serious for the patient as it could possibly have been had the ovaries and tubes been the seat of every pathological development which has ever been known to affect these organs. In fact, the results have, perhaps, been more brilliant than in any other case of this series.

I would not refuse to remove the ovaries and Fallopian tubes for the relief of a malady which could not be cured by any other means, if that malady rendered the patient a miserable and useless invalid, and if the patient could be cured by that operation.

Much disease may exist in the appendages with very little indication for their removal. A proposition which should make the extent to which the appendages are diseased proportionate to the indication for their removal would be untenable.

The operation is certainly liable to an immense amount of abuse, a kind of abuse which is most serious for the patient, and on this account it is probably becoming the subject of vigorous criticism, which should have the fortunate effect of limiting its performance to suitable cases. The removal of the appendages produces senile atrophy of the remaining reproductive organs, even though these organs be the seat of disease. Cessation of function follows, therefore, even though that function be modified by disease—that is, with the atrophic process, pathological conditions cease. Consequently a dangerous possibility of this operation lies in the fact that it is certainly capable of curing a number of maladies which ought to be relieved by other means. The temptation to produce brilliant results by a prompt and certain remedy must always be great when other measures are long, tedious, and uncertain.

